# Integration of Hollow Microneedle Arrays with Jellyfish-Shaped Electrochemical Sensor for the Detection of Biomarkers in Interstitial Fluid

**DOI:** 10.3390/s24123729

**Published:** 2024-06-08

**Authors:** Fangfang Luo, Zhanhong Li, Yiping Shi, Wen Sun, Yuwei Wang, Jianchao Sun, Zheyuan Fan, Yanyi Chang, Zifeng Wang, Yutong Han, Zhigang Zhu, Jean-Louis Marty

**Affiliations:** 1 School of Health Science and Engineering, University of Shanghai for Science and Technology, 516 Jungong Road, Shanghai 200093, China; ffluo2002@163.com (F.L.); syp2003@163.com (Y.S.); swen0813@163.com (W.S.); wangyuwei010@163.com (Y.W.); chaogege6662021@163.com (J.S.); swl3176225422@163.com (Z.F.); kusa0827@163.com (Y.C.); zfwang@usst.edu.cn (Z.W.); yutonghan@usst.edu.cn (Y.H.); zgzhu@usst.edu.cn (Z.Z.); 2 UFR Sciences, Université de Perpignan Via Domitia, 52 Avenue Paul Alduy, CEDEX, 66860 Perpignan, France; jlmarty@univ-perp.fr

**Keywords:** uric acid, glucose, pH, microneedle array, interstitial fluid

## Abstract

This study integrates hollow microneedle arrays (HMNA) with a novel jellyfish-shaped electrochemical sensor for the detection of key biomarkers, including uric acid (UA), glucose, and pH, in artificial interstitial fluid. The jellyfish-shaped sensor displayed linear responses in detecting UA and glucose via differential pulse voltammetry (DPV) and chronoamperometry, respectively. Notably, the open circuit potential (OCP) of the system showed a linear variation with pH changes, validating its pH-sensing capability. The sensor system demonstrates exceptional electrochemical responsiveness within the physiological concentration ranges of these biomarkers in simulated epidermis sensing applications. The detection linear ranges of UA, glucose, and pH were 0~0.8 mM, 0~7 mM, and 4.0~8.0, respectively. These findings highlight the potential of the HMNA-integrated jellyfish-shaped sensors in real-world epidermal applications for comprehensive disease diagnosis and health monitoring.

## 1. Introduction

With the rapid advancements in biomedical fields and the increasing individual focus on personal health status, the detection of biomarkers within the human body plays a pivotal role in disease diagnosis, prevention, and treatment. Uric acid (UA), for instance, serves as a biomarker for hyperuricemia and gout [1,2]; glucose is an indicator of diabetes or hypoglycemia [3,4,5]; pH levels are reflective of metabolic health [6] and acid-base balance [7]. Furthermore, elevated serum UA concentrations are regarded as one of the best independent predictors of diabetes, often preceding the development of insulin resistance and type 2 diabetes [8]. There are also reports of an increased prevalence of nephrolithiasis in diabetic patients, with low urinary pH being the principal factor in uric acid stone formation [9]. Consequently, glucose, uric acid, and pH, as biomarkers, are interconnected in disease diagnostics. Moreover, the pH of the medium can influence the detection of other biomarkers, such as uric acid and glucose, by affecting the acidity or alkalinity of solutions or the activity of enzymes [10]. Therefore, the concurrent measurement of glucose, uric acid, and pH is of substantial importance for comprehensive diagnostics of related diseases.

Clinically, the most prevalent biofluid sampled for health or disease monitoring-related biomarkers is blood. However, invasive blood collection typically necessitates trained medical personnel, exposes the subject to infection risks, and imposes considerable psychological stress. Alternatively, non-invasive or minimally invasive biomarker sampling offers a new avenue for health monitoring and disease diagnosis, facilitating convenient and rapid testing for related conditions [11]. This approach can empower personalized medicine, precision therapy, and individual self-health management. Extensive research has explored non-invasive or minimally invasive methods to sense biomarkers in a variety of bodily fluids, including sweat [12], tears [13], saliva [14], urine [15], and interstitial fluid (ISF) [16]. Yet, more accessible bodily fluids (e.g., sweat, tears, saliva, and urine) suffer from inconsistency in sample collection, dilution, compositional variations, and heightened susceptibility to contamination.

ISF, formed by plasma extravasation from capillaries, serves as the medium for exchanging signaling molecules, nutrients, and waste between capillaries and surrounding tissues and cells. Compared to blood, ISF resides closer to the skin surface and can be harvested painlessly or with minimal discomfort using minimally invasive techniques, without clotting or blood cell interference. ISF harbors valuable biomarkers relevant to health monitoring and disease diagnosis, such as glucose, UA, urea, lactate, cortisol, Na^+^, K^+^, and so on, whose concentrations mirror those in blood [17,18]. To a certain extent, ISF is thus seen as an alternative biofluid to blood for diagnostic purposes [19].

The extraction of ISF via microneedles for minimally invasive sampling is currently one of the popular methodologies in studying biomarkers sensing within ISF [20,21,22,23]. Typically, these microneedles, ranging in length from 500 to 1000 μm, painlessly penetrate the human stratum corneum barrier to facilitate biomarker retrieval from ISF. Over the past decades, microneedle technology has rapidly progressed from single biomarker detection to the analysis of multiple biomarkers, allowing for a more comprehensive assessment of an individual’s health status [24,25,26,27]. Conventional biomarker assays, such as the enzyme-linked immunosorbent assay (ELISA), are time-consuming, labor-intensive, and require specialized equipment or skilled operators, making them less amenable to adoption as portable personal health monitoring tools. Recently, there has been a report of the simultaneous colorimetric detection of glucose, uric acid, and pH [28]. Electrochemical sensing methods for biomarkers, on the other hand, allow for the easy miniaturization of devices and offer rapid, sensitive detection, thereby presenting a quick and convenient means for individual biomarker analysis.

This study employs 3D printing to construct a polymer hollow microneedle array (HMNA) and combines paper-cutting with printing techniques for electrode fabrication, thereby fabricating a jellyfish-shaped multifunctional electrochemical sensor. By integrating the HMNA with the electrochemical sensor, we pioneer its use for the electrochemical sensing of three biomarkers—uric acid, glucose, and pH—in interstitial fluid. This approach provides more holistic information for disease diagnosis and health management.

## 2. Experimental

### 2.1. Chemicals and Materials

The following reagents were acquired from specified suppliers: Na_2_HPO_4_·12H_2_O (≥99%), NaH_2_PO_4_·2H_2_O (≥99%), NaCl (≥99.8%), CaCl_2_ (≥96%), D(+)–glucose anhydrous (≥99.5%), sucrose (>99%), agarose, KCl (≥99.5%), FeCl_3_ (≥97%), HCl (36%~38%), K_3_Fe(CN)_6_ (≥99.5%), ascorbic acid (≥99.7%), ethanol, H_2_O_2_ (30%), acetic acid (≥99.8%), lactic acid (85–92%), NaOH (≥96%), MgSO_4_ (≥98%), and HEPES (≥99%) were all sourced from Sinopharm Chemical Reagent Co., Ltd. (Shanghai, China). Britton-Robinson buffer was purchased from Shanghai Yuanye Bio-Technology Co., Ltd. (Shanghai, China). Glucose oxidase (GOD, ≥100 U/mg) was supplied by Sangon Biotech (Shanghai, China). Uric acid (≥98%) was obtained from TCI Shanghai. The photopolymer resin was provided by Shenzhen Anycubic Technology Co., Ltd. (Shenzhen, China). Nafion (5 wt%) and chitosan (medium molecular weight) were both purchased from Sigma-Aldrich (Shanghai, China). -COOH Functionalized Multi-walled Carbon Nanotubes (MWCNT-COOH, ≥98 wt%) were sourced from Chengdu Organic Chemicals Co. Ltd. (Chengdu, China). The carbon and silver/silver chloride (60:40) inks were bought from SunChemical Co. (Parsippany, NJ, USA). A 0.1 M phosphate buffer solution (PBS) with a pH of 7.4 was prepared using Na_2_HPO_4_·12H_2_O and NaH_2_PO_4_·2H_2_O. All chemical substances were employed without undergoing any additional purification steps. Throughout the experiments, deionized water of high purity, generated by a Milli-Q system (18.2 MΩ·cm at 25 °C), was utilized.

### 2.2. Apparatus

Electrochemical evaluations were performed using a CHI-760e workstation (Austin, TX, USA). Electrode printing stencils were cut on vinyl transfer film using a Roland desktop cutter GS-24. The fabrication of hollow microneedle arrays was carried out using a PHOTON MONO X 6K 3D printer (Shenzhen Anycubic Technology Co., Ltd., Shenzhen, China). Morphological observations were conducted utilizing a ZEISS Gemini 300 scanning electron microscope (SEM, Oberkochen, Germany).

### 2.3. Preparation of Artificial Interstitial Fluid (AISF)

The artificial interstitial fluid was prepared by mixing 2.5 mM CaCl_2_, 5.5 mM glucose, 10 mM HEPES, 3.5 mM KCl, 0.7 mM MgSO_4_, 123 mM NaCl, 1.5 mM NaH_2_PO_4_, and 7.4 mM sucrose solution, with the final pH of the solution adjusted to 7.0 [29].

### 2.4. Preparation of the Electrodes

**Figure 1:**
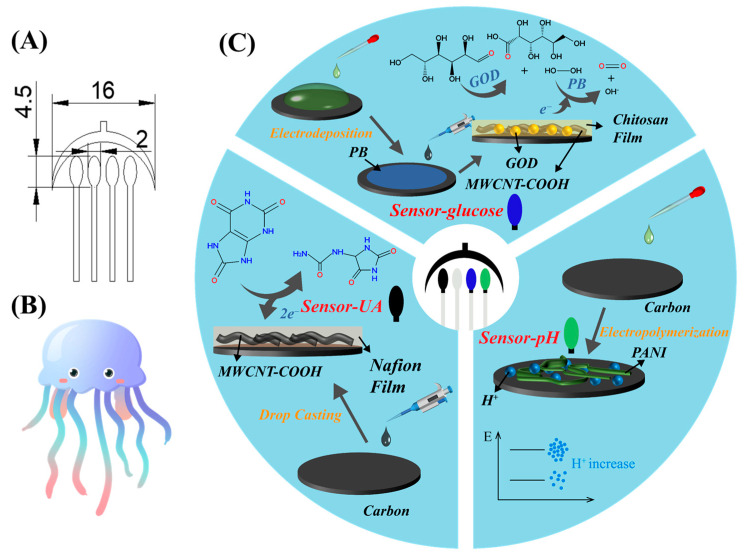
(**A**) Electrode design and dimensions (unit: mm), (**B**) which resembles a jellyfish, and (**C**) the distribution and modification of the electrodes of the jellyfish-shaped sensor, as well as the electrochemical sensing principles of each electrode.

A jellyfish-shaped electrode printing stencil was designed using CAD software (AutoCAD 2021), with the electrode design and dimensions depicted in Figure 1A. Figure 1B illustrates the electrode’s resemblance to a jellyfish-shape. The corresponding electrode stencils were then cut onto a vinyl transfer film using Roland desktop cutter. Subsequently, the stencil was affixed onto a PET board, upon which the electrodes were sequentially printed using appropriate inks. The wire section connecting to the electrochemical workstation is composed of 1.3 mm wide Ag/AgCl, while the reference electrode is also made of Ag/AgCl. Both the working and counter electrodes are carbon-based. The geometric area of each working electrode, as well as that of the reference electrode, was 7.065 mm^2^. After applying each type of ink, the PET board was placed in a 60 °C oven for a 20-min drying period.

The distribution and modification of the electrodes of the jellyfish-shaped sensor, as well as the electrochemical sensing principles of each electrode, are illustrated as shown in Figure 1C.

### 2.5. Preparation of Hollow Microneedle Array (HMNA)

The HMNA was modeled using RHINO 8 3D software (Version 8.2), followed by 3D printing using a PHOTON MONO X 6K 3D printer (https://store.anycubic.com/products/photon-mono-x-6k, accessed on 25 April 2024). The array comprised 6 × 8 microneedles, each with a pyramidal shape, a height of 800 μm and a hollow microchannel diameter of 100 μm. Upon completion of printing, the HMNA underwent demolding treatment and was immersed in ethanol (95%) for ultrasonic cleaning for 3 min to remove uncured resin. Finally, the HMNA was subjected to ultraviolet cleaning in a UV cleaner for 10 min to finalize the shape.

**Figure 2:**
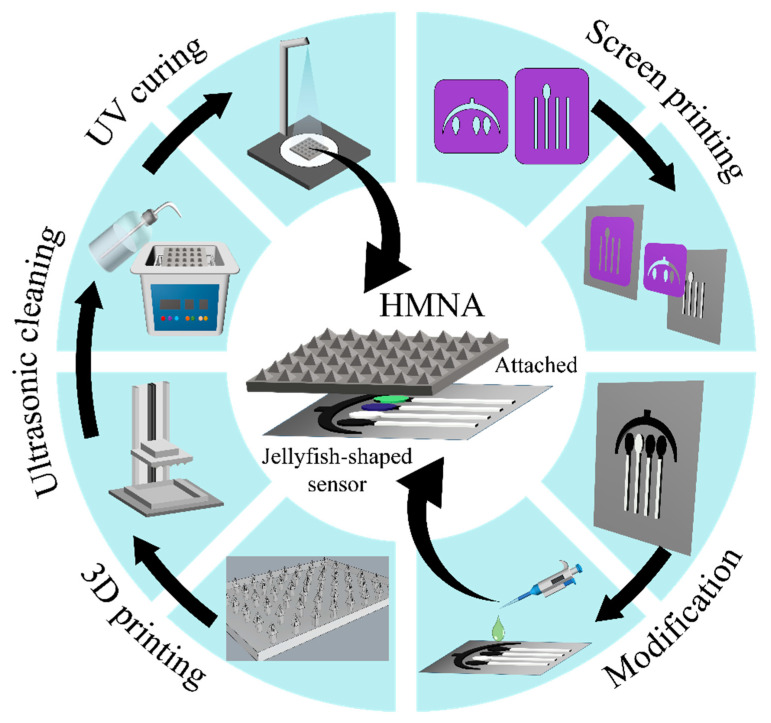
The schematic diagram of the electrode printing, HMNA 3D printing, and their coupled testing configuration.

The electrode printing, HMNA 3D printing, and their coupled testing configuration are illustrated in Figure 2. In the test, the sensors were in direct contact with the back side of HMNA, where the HMNA served to extract ISF while the sensors promptly detected the biomarkers from the ISF sampled by the HMNA.

### 2.6. Preparation of the Sensor

#### 2.6.1. Preparation of Sensor-UA

The preparation method for the Sensor-UA is similar to our previous work [24]. Specifically, a 5 wt% Nafion solution was first diluted with deionized water to obtain a 2 wt% Nafion solution. Subsequently, 1 mg of MWCNT-COOH was mixed with 1 mL of the 2 wt% Nafion solution under ultrasonic assistance for half an hour. Finally, 4 µL of the MWCNT-COOH/Nafion mixture was drop-cast onto a carbon working electrode. After natural air-drying, the resulting sensor was designated Sensor-UA.

#### 2.6.2. Preparation of Sensor-Glucose

First, Prussian Blue (PB) was electrodeposited onto an unmodified carbon working electrode. The procedure involved adding a mixture of 2.5 mM FeCl_3_, 2.5 mM K_3_[Fe(CN)_6_], 100 mM KCl, and 100 mM HCl onto the electrode surface, followed by performing 10 cyclic voltammetry (CV) cycles at a scan rate of 50 mV/s within the potential range of −0.2 V to 0.6 V, thus depositing PB. After rinsing the modified electrode with deionized water, it was placed in an oven at 90 °C for 4 h. Figure S1A presents the cyclic voltammogram of PB deposition.

Subsequently, GOD was immobilized onto the PB-modified electrode. This was accomplished by dispersing 2 mg MWCNT-COOH in 1 mL of a mixture containing 1 wt% acetic acid and 2 wt% chitosan, followed by mechanically stirring overnight to yield an MWCNT-COOH/chitosan mixture. Then, GOD was dissolved in PBS to prepare a 40 mg/mL GOD solution. The GOD solution was then mixed with the MWCNT-COOH/chitosan mixture at a volume ratio of 1:2, forming a GOD/MWCNT-COOH/chitosan composite. Finally, 4 µL of the GOD/MWCNT-COOH/chitosan composite was drop-cast onto the PB-modified electrode, which was then incubated overnight in a refrigerator at 4 °C. The prepared sensor was designated Sensor-glucose.

#### 2.6.3. Preparation of Sensor-pH

First, 0.5 M HCl was drop-cast onto the surface of an unmodified carbon electrode. Subsequently, 10 CV cycles were performed within the potential range of −0.1 V to 0.9 V at a scan rate of 0.1 V/s to electrochemically clean the electrode. After rinsing the electrode with deionized water, a mixture containing 0.1 M aniline and 1 M HCl was added onto the electrode surface. Under the potential range of −0.2 V to 1.0 V and a scan rate of 0.1 V/s, 12 CV cycles were conducted on the electrode to facilitate the electropolymerization of polyaniline. The modified electrode was then rinsed again with deionized water and designated Sensor-pH. Figure S1B displays the CV curves of aniline electropolymerization.

## 3. Results and Discussion

### 3.1. SEM Characterization

**Figure 3:**
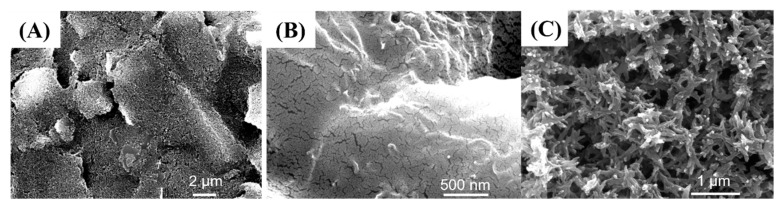
The SEM characterization of the modified electrodes: (**A**) unmodified carbon electrode, (**B**) MWCNT-COOH/Nafion-mixture-modified electrode, and (**C**) electropolymerized polyaniline modified electrode.

To observe the surface morphology of each modified electrode of the sensor, we characterized the electrodes using SEM. As shown in Figure 3A, the unmodified carbon electrode exhibited a stacked lamellae structure. The surface of the lamellar was rough, formed by numerous closely packed carbon particles, ensuring the electrical conductivity of the substrate electrode for the sensor. Figure 3B presents the SEM image of the MWCNT-COOH/Nafion-mixture-modified electrode, clearly revealing a distinct Nafion film covering the electrode surface. The Nafion film served to prevent the detachment of MWCNT-COOH from the electrode. Despite the MWCNT-COOH being effectively encapsulated by the Nafion membrane, the modified electrode surface still retained the characteristic fibrous structure of MWCNT-COOH. Figure 3C displays the surface structure of the electropolymerized polyaniline. It demonstrates that the electrode surface is composed of fibrous polyaniline, the typical feature of the morphology of electropolymerized polyaniline [30].

### 3.2. Electrochemical Performance of the Jellyfish-Shaped Sensor

#### 3.2.1. Sensor-UA

**Figure 4:**
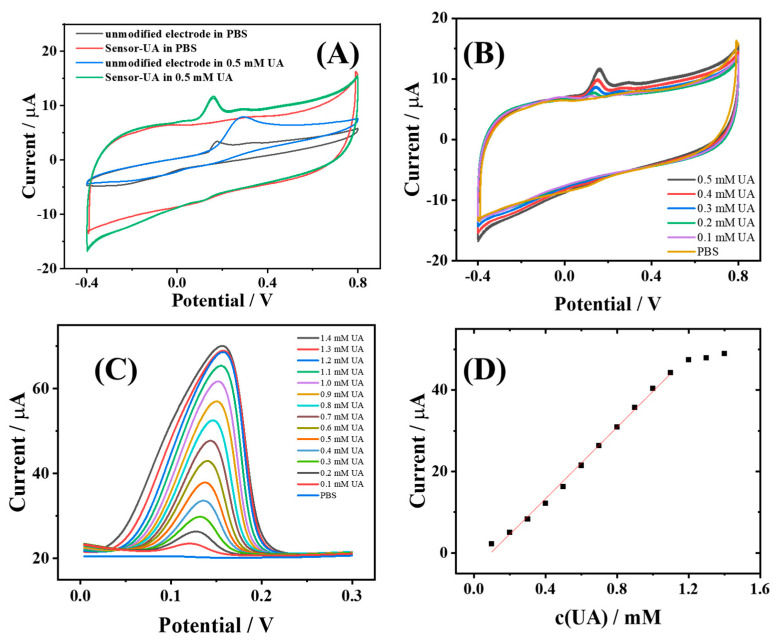
The electrochemical characterization of Sensor-UA: (**A**) CVs of unmodified and MWCNT-COOH-modified carbon electrodes in 0.1M PBS, pH 7.4, in the absence and presence of 0.5 mM UA. Potential range −0.4 V to 0.8 V; scan rate 50 mV/s. (**B**) CVs of Sensor-UA with different concentrations of UA in PBS. UA concentrations were 0, 0.1, 0.2, 0.3, 0.4, and 0.5 mM, respectively. Potential range −0.4 V to 0.8 V; scan rate 50 mV/s. (**C**) DPV responses of Sensor-UA to different concentrations of UA from 0.1 to 1.4 mM. Potential range 0 V to 0.3 V; amplitude 0.05 V; pulse width 0.05 s. (**D**) The linear fitting curve of calibration for Sensor-UA’s DPV current response towards UA.

To characterize the electrochemical performance of Sensor-UA, the CV behaviors of UA on the electrode before and after modification with MWCNT-COOH were initially examined; the results are depicted in Figure 4A. It can be observed that an oxidative peak at approximately 0.17 V for the unmodified MWCNT-COOH electrode in PBS is likely due to the electrochemical oxidation of inherent impurities present in the commercial carbon ink. Upon the addition of 0.5 mM UA, an oxidative peak corresponding to UA appeared at 0.29 V for the unmodified carbon electrode, which should be attributed to the electrochemical oxidation of UA, involving the transfer of two electrons on the electrode surface, leading to the formation of allantoin. For comparison, the electrochemical CV behavior of the MWCNT-COOH-modified carbon electrode (Sensor-UA) in PBS was then investigated. As is evident from the figure, the electric double layer of the Sensor-UA broadened relative to that of the unmodified carbon electrode. This should be due to the increase in the electrode surface area resulting from the MWCNT-COOH modification. Moreover, the oxidative peak associated with impurities that was observed at around 0.17 V for the unmodified electrode disappeared. This is presumably because the substantial increase in electrode surface area caused by the introduction of MWCNT-COOH generating an augmented electric double-layer current that masked the electrochemical oxidation peak of impurities present in the unmodified carbon electrode. Subsequently, Sensor-UA was spiked with 0.5 mM UA in the solution, and an oxidative peak at 0.16 V was revealed. This can also be assigned to the electrochemical oxidation of UA. Notably, the oxidative peak potential of UA on Sensor-UA was lower than that on the unmodified carbon electrode, indicating that the modification with MWCNT-COOH enabled Sensor-UA to effectively reduce the electrochemical oxidation potential of UA, thereby exhibiting a superior UA electrochemical oxidation performance.

To further explore the sensing potential of Sensor-UA towards UA, we tested its CV-response characteristics upon the addition of different concentrations of UA, as illustrated in Figure 4B. It is evident that as the concentration of UA was increased stepwise from 0 to 0.5 mM, the oxidative peak current of Sensor-UA, corresponding to UA, also increased, accompanied by a progressive shift in the peak potential towards more positive values. The gradual positive shift in the electrochemical oxidation peak with increasing UA concentration may result from a combination of mass transport effects, changes in electrode surface coverage, and the electrode local environment. These observations substantiate the electrochemical sensing capability of Sensor-UA for UA.

Differential pulse voltammetry (DPV) can be utilized effectively to subtract background currents from the sensor, enabling the acquisition of pronounced current response information corresponding to target species concentrations. To assess the electrochemical sensing performance of Sensor-UA towards UA, DPV was employed to investigate the sensor’s response to varying UA concentrations, as shown in Figure 4C. With the UA concentration incrementally increasing from 0 to 1.4 mM, the DPV response peak current of Sensor-UA for UA steadily rose. The calibration curve depicting this response is presented in Figure 4D. It can be deduced that Sensor-UA exhibited a linear response range for UA spanning from 0.1 to 1.2 mM, with a sensitivity of 43.81 μA/mM and a detection limit of 0.01 μM (S/N = 3). The linear fitting equation for this calibration curve is as follows:

I (μA) = 43.81 × c(UA) (mM) − 4.18 (μA) (R^2^ = 0.9956)

The results of the reusability and anti-interference performance tests for Sensor-UA are depicted in Figure S2, showcasing its excellent reusability and robust anti-interference capabilities.

#### 3.2.2. Sensor-Glucose

**Figure 5:**
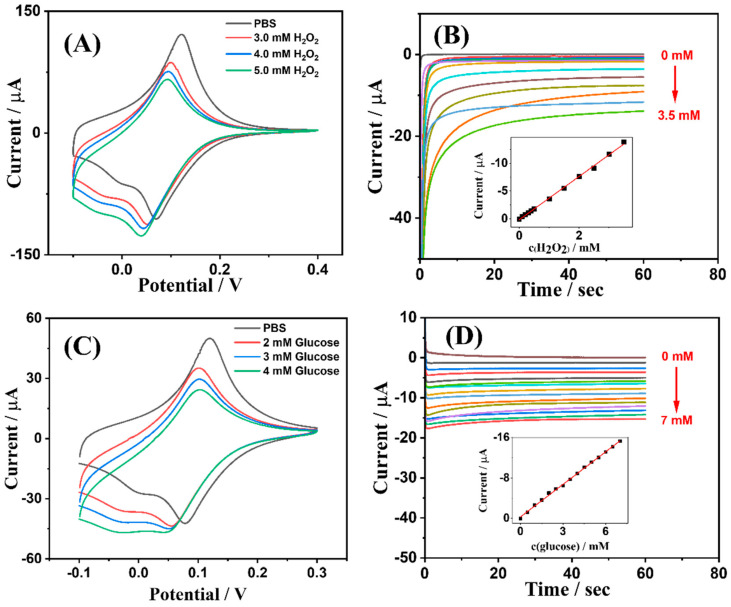
The electrochemical characterization of Sensor-Glucose: (**A**) CVs of PB-modified electrode in 0.1M PBS + 0.1 M KCl, pH 7.4, containing different concentrations of H_2_O_2_. Potential range −0.1 V to 0.4 V; scan rate 50 mV/s. (**B**) Chronoamperometric response of PB-modified electrode in 0.1M PBS + 0.1 M KCl, pH 7.4, to different concentrations of H_2_O_2_. The concentrations of H_2_O_2_ were 0.1, 0.2, 0.3, 0.4, 0.5, 1.0, 1.5, 2.0, 2.5, 3.0, and 3.5 mM, respectively. Applied potential 0.07 V; applied time 60 s. The inset shows the linear fitting calibration curve. (**C**) CVs of Sensor-Glucose in 0.1M PBS, pH 7.4, containing different concentrations of glucose. Potential range −0.1 V to 0.3 V; scan rate 50 mV/s. (**D**) Chronoamperometric response of Sensor-Glucose in 0.1M PBS, pH 7.4, to different concentrations of glucose. The concentrations of glucose were 0.5, 1.0, 1.5, 2.0, 2.5, 3.0, 3.5, 4.0, 4.5, 5.0, 5.5, 6.0, 6.5, and 7.0 mM, respectively. Applied potential 0.07 V; applied time 60 s. The inset shows the linear fitting calibration curve.

In the electrochemical sensing process of Sensor-Glucose for glucose, glucose is first enzymatically oxidized by GOD in the presence of oxygen, producing gluconic acid and H_2_O_2_. The generated H_2_O_2_ is subsequently electrochemically reduced at the PB-modified electrode. The magnitude of this current is directly proportional to the glucose concentration [31]. Thus, to investigate the glucose electrochemical sensing performance of Sensor-Glucose, the H_2_O_2_ electrochemical sensing performance of the PB-modified electrode was first examined. As shown in Figure 5A, it presents cyclic voltammograms of the PB-modified electrode for various concentrations of H_2_O_2_. The concentrations of H_2_O_2_ are indicated in the figure. In PBS solution without H_2_O_2_, the PB-modified electrode exhibited a well-defined pair of reversible oxidation-reduction peaks, which corresponded to the redox transitions between Fe(II) and Fe(III) within PB. The oxidation-reduction potentials were 0.12 V and 0.07 V, respectively. The difference between them was 0.05 V, or 50 mV, which was close to the theoretical value of 59 mV for a single-electron transfer process according to the Nernst equation, thus demonstrating the excellent electrochemical reversibility of the PB-modified electrode. Moreover, with increasing H_2_O_2_ concentration, the oxidative peak of PB gradually decreased while the reductive peak grew, substantiating the electrochemical reduction of H_2_O_2_ occurring at the PB-modified electrode [32].

Figure 5B shows the chronoamperometric response curves of the PB-modified electrode for different concentrations of H_2_O_2_. As the H_2_O_2_ concentration increased, the electrochemical reduction current of the PB-modified electrode steadily rose. The inset depicts a plot of current response intensity versus H_2_O_2_ concentration. It can be seen that the sensor exhibited a good linear response range for H_2_O_2_ from 0.1 to 3.5 mM, with a sensitivity of 3.89 μA/mM and a detection limit of 1.09 μM (S/N = 3). The linear regression equation for this curve is as follows:

I (μA) = −3.89 × c(H_2_O_2_) (mM) + 0.16 (μA) (R^2^ = 0.9979)

Figure 5C presents the electrochemical CVs testing of Sensor-Glucose for different glucose concentrations. The concentrations of glucose are indicated in the figure. In the absence of glucose, the CV curve of Sensor-Glucose in PBS exhibited a well-defined pair of reversible electrochemical oxidation-reduction peaks, which corresponded to the electrochemical redox behavior of PB. Similar to the discussion concerning the PB-modified electrode above, the electrochemical oxidation and reduction peaks for Sensor-Glucose in PBS were also found at 0.12 V and 0.07 V, respectively, with the difference between them close to the theoretical value for a single-electron transfer process according to the Nernst equation. This indicated that the state of the electrode was not significantly altered following modification with the GOD/MWCNT-COOH/chitosan membrane. Moreover, upon the addition of glucose, similar to the results discussed for the PB-modified electrode, the electrochemical oxidation peak of Sensor-Glucose gradually diminished, while the electrochemical reduction peak increased. This confirmed the process by which glucose was enzymatically oxidized on Sensor-Glucose to generate H_2_O_2_, which was then electrochemically reduced at the PB-modified electrode.

Figure 5D displays the chronoamperometric response curves of Sensor-Glucose for varying glucose concentrations. It is evident that the electrochemical current of the sensor continuously increased with escalating glucose concentrations, substantiating the feasibility of glucose electrochemical sensing using this sensor. The inset shows the calibration curve for the sensor’s response to different glucose concentrations. The fitted linear range is 0.5 to 7 mM, with a sensitivity of 2.15 μA/mM, and a detection limit of 1.71 μM. The regression equation for this curve is as follows:

I (μA) = −2.15 × c(glucose) (mM) − 0.28 (μA) (R^2^ = 0.9984)

The results of the reusability and anti-interference performance tests for Sensor-Glucose are depicted in Figure S3, showcasing its excellent reusability and robust anti-interference capabilities.

#### 3.2.3. Sensor-pH

**Figure 6:**
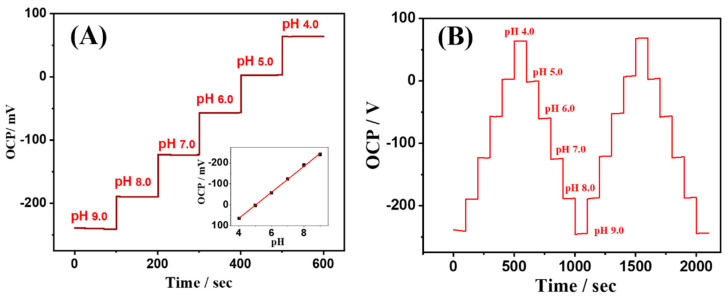
(**A**) The open-circuit potential response of the Sensor-pH to different pH values. The inset shows the linear relationship between the sensor’s OCP and the measured pH. Each OCP for pH was measured for 100 s. (**B**) Repeatability of the Sensor-pH when the pH of the solution undergoes continuous alterations.

To obtain different pH values of the tested solution, 0.2 M NaOH was added to the Britton–Robinson buffer solution to adjust the pH to 4.0, 5.0, 6.0, 7.0, 8.0, and 9.0, respectively. The open-circuit potential (OCP) of the Sensor-pH was tested in each of these solutions, with each OCP measurement lasting for 100 s. As shown in Figure 6A, the results indicate that as the pH decreased from 9.0, the OCP of the Sensor-pH rose from −240 mV, reaching 64 mV at pH = 4.0. The inset demonstrates a linear relationship between the sensor’s OCP and the measured pH, with a slope of −60.5 mV/pH, closely approaching the theoretical value of −59 mV/pH predicted by the Nernst equation for OCP–pH relationship.

To evaluate the repeatability of the Sensor-pH, the sensor was immersed in solutions with varying pH values ranging from 9.0 down to 4.0, after which the pH of the solutions was raised back up to 9.0, and the OCP of the Sensor-pH was measured across all the solutions. The results, depicted in Figure 6B, show that the Sensor-pH exhibited nearly identical OCP responses before and after each pH change, indicating the excellent reusability and repeatability of the sensor under continuous alterations in the solution pH.

### 3.3. Electrochemical Sensing for Artificial Interstitial Fluid Samples

**Figure 7:**
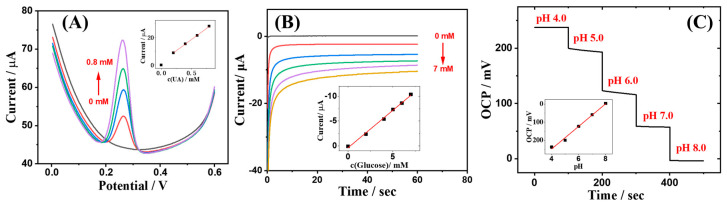
The electrochemical sensing performance of the jellyfish-shaped sensor. (**A**) DPVs of the sensor to UA. The concentration of UA was 0, 0.2, 0.4, 0.6, and 0.8 mM, respectively. Potential range 0 to 0.6 V; amplitude 0.05 V; pulse width 0.05 s. (**B**) Chronoamperometric response of the sensor to glucose. The concentration of glucose was 0, 2, 4, 5, 6, and 7 mM, respectively. Potential applied 0.07 V; applied time 60 s. (**C**) OCP response of the sensor to different values of pH. The pH value tested is indicated in the figure.

To assess the practical application potential of the jellyfish-shaped sensor, its electrochemical sensing performance for biomarkers in AISF was initially examined. As shown in Figure 7A, upon immersion of the sensor in AISF and the subsequent addition of varying concentrations of uric acid, the sensor demonstrated a favorable electrochemical response to uric acid within a concentration range of 0 to 0.8 mM via DPV. Moreover, a conspicuous electrochemical oxidation peak appeared at a potential of 0.27 V. The inset illustrates a calibration curve of peak current versus uric acid concentration, revealing a strong linear relationship with a response slope of 32.05 μA/mM and an R^2^ value of 0.9996. 

Similarly, the electrochemical sensing capability of glucose was evaluated. As depicted in Figure 7B, an increase in the reduction current response was observed as the glucose concentration rose. The inset presents the relationship between the current response and glucose concentration over the range of 0 to 7 mM, evidencing a good linear response of the sensor to glucose concentrations, with a slope of −1.51 μA/mM and an R^2^ value of 0.9964. 

Lastly, the OCP response of the jellyfish-shaped sensor to a pH range of 4.0~8.0 in the AISF was tested, as shown in Figure 7C, where the sensor exhibited a discernible stepwise response to different pH levels. The inset figure displays the linear correlation between the obtained OCP and pH, with a slope of −62.2 mV/pH, closely approximating the theoretical value predicted by the Nernst equation for the OCP–pH relationship. 

Collectively, the sensor demonstrates an excellent sensing performance to uric acid, glucose, and pH in AISF. It is worth mentioning that the sensor’s sensing range covered the physiological concentration ranges of these three biomarkers (UA: 0.12~0.45 mM [18], glucose: 3.6~6.0 mM [18,33], pH: 6.60~7.60 [20]). These findings underscore the immense application potential of this sensor.

### 3.4. Electrochemical Sensing in Simulated Epidermis

**Figure 8:**
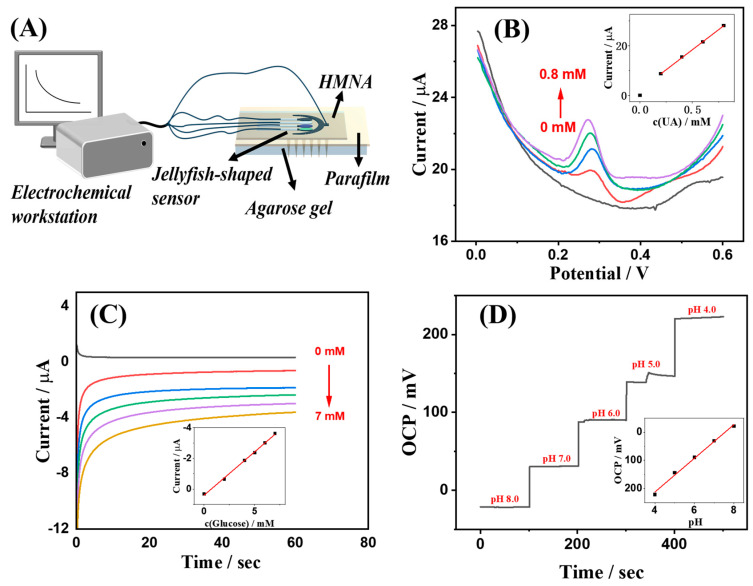
HMNA-integrated jellyfish-shaped electrochemical sensing system for biomarker sensing in simulated epidermis. (**A**) The schematic diagram of the experimental setup for the test. (**B**) DPVs for UA sensing. The concentration of UA was 0, 0.2, 0.4, 0.6, and 0.8 mM, respectively. Potential range 0 to 0.6 V; amplitude 0.05 V; pulse width 0.05 s. (**C**) Chronoamperometric response for glucose sensing. The concentration of glucose was 0, 2, 4, 5, 6, and 7 mM, respectively. Potential applied 0.07 V; applied time 60 s. (**D**) OCP response for pH sensing. The pH value tested is indicated in the figure.

To evaluate the stratum corneum penetration and electrochemical sensing performance of biomarkers of the HMNA-integrated jellyfish-shaped electrochemical sensor system, a puncture experiment was conducted using parafilm to simulate the human stratum corneum and a 5 wt% agarose hydrogel to mimic epidermis. The schematic diagram of the experimental setup for the test is shown in Figure 8A. The preparation of 5 wt% agarose hydrogel was as follows: 1 g agarose was dissolved in 20 mL deionized water, and the mixture was heated to 105 °C until the agarose was fully dissolved, yielding a clear transparent solution, which was then allowed to cool to room temperature for use. In the experiment, a certain pressure applied by a finger on the backside of the HMNA enabled it to easily penetrate the simulated stratum corneum. Subsequently, the jellyfish-shaped electrochemical sensor was attached to the back of the HMNA to conduct the corresponding electrochemical measurements. During the testing process, AISF containing biomarkers with appropriate concentrations was added beneath the simulated epidermis for electrochemical sensing.

Figure 8B illustrates the electrochemical sensing results of the sensing system for uric acid, where it is evident that the DPV peak current intensity of the sensing system for uric acid concentrations within the physiological ranges beneath the simulated epidermis increased with rising uric acid levels. The inset displays a calibration curve of peak currents from DPV responses to uric acid concentrations, exhibiting a strong linear correlation. 

Figure 8C depicts the electrochemical sensing outcomes for glucose within the simulated epidermis, revealing a proportional relationship between the system’s current response and glucose concentrations under physiological conditions. The inset shows the relationship between response currents and glucose concentration, also demonstrating good linearity. 

Figure 8D presents the pH-sensing results of the system in the simulated epidermis, with the OCP of the sensing system varying linearly as the pH shifted from 4.0 to 8.0. The inset provides a linear relationship graph of OCP versus pH. 

Collectively, these results confirm that the HMNA-integrated jellyfish-shaped electrochemical sensor system demonstrated excellent electrochemical responsiveness to uric acid, glucose, and pH under simulated epidermal conditions, exhibiting favorable linear responses for the physiological concentrations of these biomarkers. This validates the potential application of this sensing system in real epidermal scenarios.

## 4. Conclusions

In this study, we developed and validated an HMNA-integrated jellyfish-shaped electrochemical sensor system capable of concurrently measuring uric acid, glucose, and pH in a simulated epidermal. The system’s sensitivity and linearity across physiological concentrations affirm its viability for the monitoring of biomarkers related to diabetes, gout, and metabolic health. The sensor’s design overcomes the challenges associated with conventional sampling methods and paves the way for advanced wearable health monitoring technologies. By enabling the real-time and multiplexed analysis of critical biomarkers, this innovation contributes to the advancement of personalized medicine and facilitates early disease detection and management.

## Data Availability

Data are contained within the article and Supplementary Materials.

## Supplementary Materials

The following supporting information can be downloaded at: https://www.mdpi.com/article/10.3390/s24123729/s1, Figure S1: The cyclic voltammograms of (A) PB electrodeposition, and (B) polyaniline electropolymerization; Figure S2: The reusability and anti-interference performance tests for Sensor-UA. DPV tests: Potential range 0 V to 0.3 V, amplitude 0.05 V, pulse width 0.05 s. (A) The sensor was scanned for 10 times in PBS, pH 7.4, containing 0.5 mM UA. (B) Anti-interference test of the Sensor-UA for common physiological interferences. Glu: glucose; AA: ascorbic acid; Cr: Creatinine; LA: lactic acid; Figure S3: The reusability and anti-interference performance tests for Sensor-glucose. Chronoamperometric tests: Applied potential 0.07 V, applied time 60 s. (A) The sensor was scanned for 14 times in PBS, pH 7.4, containing 2.5 mM glucose. (B) Anti-interference test of the Sensor-glucose for common physiological interferences. UA: uric acid; AA: ascorbic acid; Cr: Creatinine; LA: lactic acid.
