# Supplementary material for: Integration of Hollow Microneedle Arrays with Jellyfish-Shaped Electrochemical Sensor for the Detection of Biomarkers in Interstitial Fluid
Source: Sensors (Basel). 2024 Jun 8;24(12):3729. doi: 10.3390/s24123729 (PMC11207310; doi:10.3390/s24123729)
Supplement: Supplementary file 1 [file sensors-24-03729-s001.zip › sensors-3011854-supplementary.pdf]

Supplementary Data

Integration of Hollow Microneedle Arrays with Jellyfish-Shaped Electrochemical Sensor for the Detection of Biomarkers in Interstitial Fluid

Fangfang Luo [1], Zhanhong Li [1,*], Yiping Shi [1], Wen Sun [1], Yuwei Wang [1], Jianchao Sun [1], Zheyuan Fan [1], Yanyi Chang [1], Zifeng Wang [1], Yutong Han [1], Zhigang Zhu [1] and Jean-Louis Marty [2]

1 School of Health Science and Engineering, University of Shanghai for Science and Technology, 516 Jungong Road, Shanghai 200093, China; ffluo2002@163.com (F.L.); syp2003@163.com (Y.S.); swen0813@163.com (W.S.); wangyuwei010@163.com (Y.W.); chaogege6662021@163.com (J.S.); swl3176225422@163.com (Z.F.); kusa0827@163.com (Y.C.); zfwang@usst.edu.cn (Z.W.); yutonghan@usst.edu.cn (Y.H.); zgzhu@usst.edu.cn (Z.Z.)

2 UFR Sciences, Université de Perpignan Via Domitia, 52 Avenue Paul Alduy, 66860 Perpignan CEDEX, France; jlmarty@univ-perp.fr

* Correspondence: zhli@usst.edu.cn

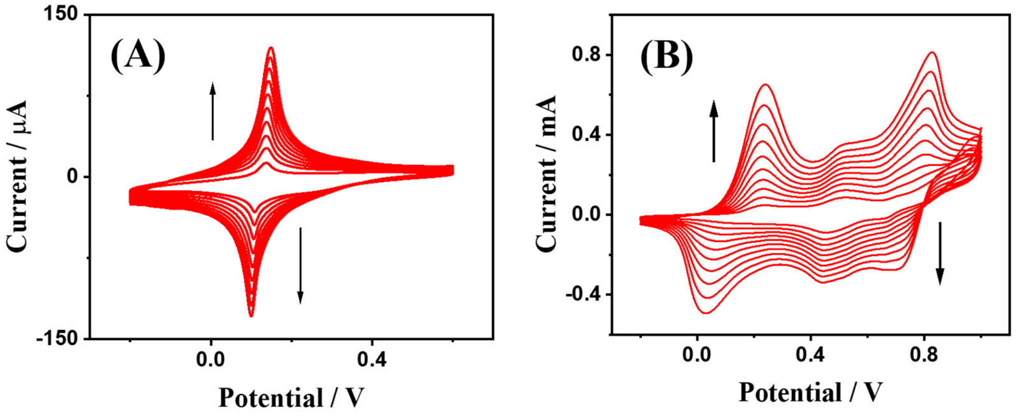

Figure S1. The cyclic voltammograms of (**A**) PB electrodeposition, and (**B**) polyaniline electropolymerization.

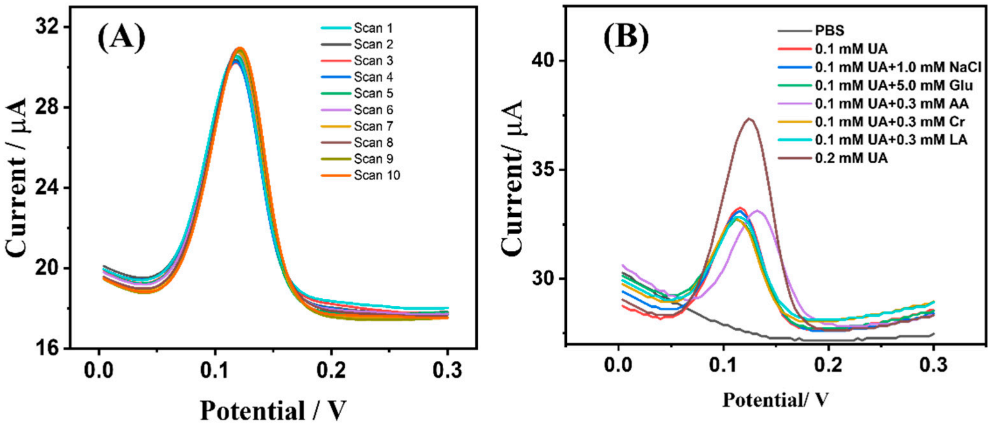

Figure S2. The reusability and anti-interference performance tests for Sensor-UA. DPV tests: Potential range 0 V to 0.3 V, amplitude 0.05 V, pulse width 0.05 s. (**A**) The sensor was scanned for 10 times in PBS, pH 7.4, containing 0.5 mM UA. (**B**) Anti-interference test of the Sensor-UA for common physiological interferences. Glu: glucose; AA: ascorbic acid; Cr: Creatinine; LA: lactic acid.

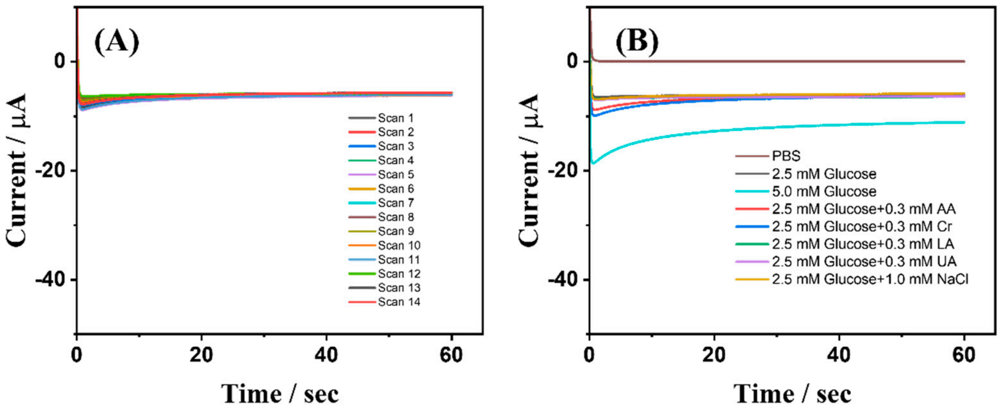

Figure S3. The reusability and anti-interference performance tests for Sensor-glucose. Chronoamperometric tests: Applied potential 0.07 V, applied time 60 s. (**A**) The sensor was scanned for 14 times in PBS, pH 7.4, containing 2.5 mM glucose. (**B**) Anti-interference test of the Sensor-glucose for common physiological interferences. UA: uric acid; AA: ascorbic acid; Cr: Creatinine; LA: lactic acid.
